# Ethyl 2-(4-nitro­phen­yl)-1-[3-(2-oxopyrrolidin-1-yl)prop­yl]-1*H*-benzimidazole-5-carboxyl­ate

**DOI:** 10.1107/S1600536811052408

**Published:** 2011-12-10

**Authors:** Yeong Keng Yoon, Mohamed Ashraf Ali, Tan Soo Choon, Safra Izuani Jama Asik, Ibrahim Abdul Razak

**Affiliations:** a Institute for Research in Molecular Medicine, Universiti Sains Malaysia, Minden 11800, Penang, Malaysia; b School of Physics, Universiti Sains Malaysia, 11800 USM, Penang, Malaysia

## Abstract

In the title compound, C_23_H_24_N_4_O_5_, the essentially planar benzimidazole ring system [maximum deviation = 0.008 (2) Å] forms a dihedral angle of 39.22 (7)° with the attached nitro­benzene ring. The pyrrolidin-2-one ring adopts an envelope conformation with a C atom as the flap. In the crystal, mol­ecules are connected by C—H⋯O inter­actions, forming sheets propagating in (011). The crystal packing also features weak π–π stacking inter­actions [centroid–centroid = 3.6746 (12) Å].

## Related literature

For applications of benzimidazole compounds, see: Rao *et al.* (2002 ▶); Ali *et al.* (2007 ▶). For the stability of the temperature controller used in the data collection, see: Cosier & Glazer (1986 ▶). For ring conformations, see: Cremer & Pople (1975 ▶).
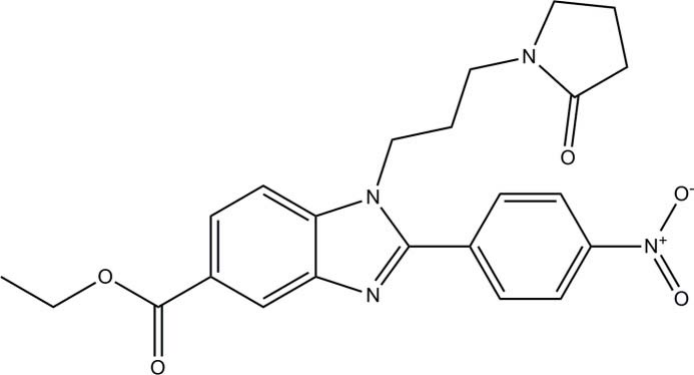


## Experimental

### 

#### Crystal data

C_23_H_24_N_4_O_5_
*M* *_r_* = 436.46
Triclinic, 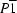

*a* = 9.3125 (1) Å
*b* = 10.0941 (1) Å
*c* = 12.9955 (2) Å
α = 91.958 (1)°
β = 107.752 (1)°
γ = 114.465 (1)°
*V* = 1040.67 (2) Å^3^
*Z* = 2
Mo *K*α radiation
μ = 0.10 mm^−1^
*T* = 100 K
0.34 × 0.20 × 0.13 mm

#### Data collection

Bruker SMART APEXII CCD diffractometer
Absorption correction: multi-scan (*SADABS*; Bruker, 2009 ▶) *T*_min_ = 0.967, *T*_max_ = 0.987
27734 measured reflections
7466 independent reflections
5375 reflections with *I* > 2σ(*I*)
*R*_int_ = 0.032

#### Refinement

*R*[*F* ^2^ > 2σ(*F* ^2^)] = 0.069
*wR*(*F* ^2^) = 0.197
*S* = 1.04
7466 reflections
289 parameters
H-atom parameters constrained
Δρ_max_ = 0.50 e Å^−3^
Δρ_min_ = −0.30 e Å^−3^

### 

Data collection: *APEX2* (Bruker, 2009 ▶); cell refinement: *SAINT* (Bruker, 2009 ▶); data reduction: *SAINT*; program(s) used to solve structure: *SHELXTL* (Sheldrick, 2008 ▶); program(s) used to refine structure: *SHELXTL*; molecular graphics: *SHELXTL*; software used to prepare material for publication: *SHELXTL* and *PLATON* (Spek, 2009 ▶).

## Supplementary Material

Crystal structure: contains datablock(s) global, I. DOI: 10.1107/S1600536811052408/hb6545sup1.cif

Structure factors: contains datablock(s) I. DOI: 10.1107/S1600536811052408/hb6545Isup2.hkl

Supplementary material file. DOI: 10.1107/S1600536811052408/hb6545Isup3.cml

Additional supplementary materials:  crystallographic information; 3D view; checkCIF report

## Figures and Tables

**Table 1:** Hydrogen-bond geometry (Å, °)

*D*—H⋯*A*	*D*—H	H⋯*A*	*D*⋯*A*	*D*—H⋯*A*
C15—H15*A*⋯O3^i^	0.99	2.41	3.355 (3)	159
C15—H15*B*⋯O3^ii^	0.99	2.38	3.186 (3)	139
C19—H19*A*⋯O2^iii^	0.99	2.38	3.312 (3)	156

Symmetry codes: (i) 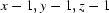; (ii) 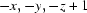; (iii) 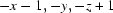.

**Fig. 1.**
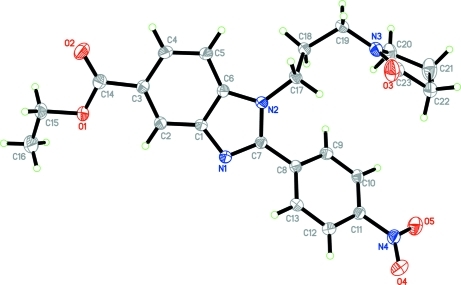
The structure of the title compound, showing 50% probability displacement ellipsoids.

**Fig. 2.**
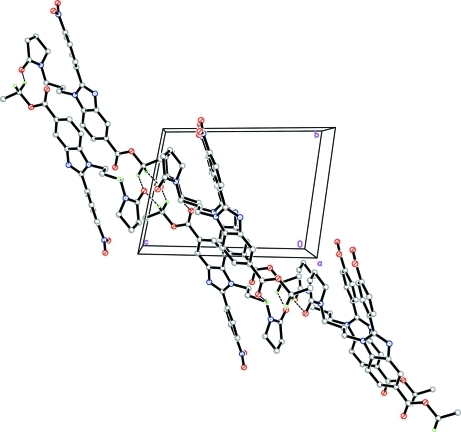
The crystal packing, viewed along the a-axis, showing two-dimensional planes parallel to (011). Hydrogen atoms that not involved in hydrogen bonding (dashed lines) are omitted for clarity.

## supplementary crystallographic information

### Comment

Benzimidazole derivatives have many clinical applications (Rao *et al.*, 2002) and are being currently evaluated for their anti-TB activities (Ali *et al.*, 2007).

In the title compound, (I), Fig. 1, the benzimidazole (N1–N2/C1–C7) ring is essentially planar with maximum deviation of 0.008 (2) for atom C3. This ring also makes dihedral angles of 77.02 (11) and 39.22 (7)° with the mean plane of pyrrolidin-2-one, (O3/N3/C20–C23) and nitrobenzene, (C8–C13) groups, respectively. The mean plane of pyrrolidin-2-one, (O3/N3/C20–C23) adopts an envelope conformation with puckering parameters Q = 0.237 (3) Å and φ = 76.0 (6)° (Cremer & Pople, 1975).

In the crystal (Fig. 2), the molecules are connected by C15—H15A···O3, C15—H15B···O3 and C19—H19A···O2 interactions to form (011) sheets. π–π stacking interactions are observed between the benzene (C1–C6; centroid *Cg*3) rings with their centroids distance of 3.6746 (12) Å [symmetry code : -x,-y,1-z].

### Experimental

Ethyl 3-amino-4-(3(2-oxopyrrolidin-1yl)propylamino)benzoate (0.84 mmol) and sodium metabisulfite adduct of 4-nitrobenzaldehyde (1.68 mmol) were dissolved in DMF. The reaction mixture was reflux at 130 °C for 2 hrs. After completion, the reaction mixture was diluted in ethyl acetate (20 ml) and washed with water (20 ml). The organic layer was collected, dried over Na_2_SO_4_ and the evaporated in *vacuo* to yield the product. The product was recrystallised from ethyl acetate as orange blocks.

### Refinement

All the H atoms were positioned geometrically and refined using a riding model with with C–H = 0.95–0.99 Å. The *U*_iso_ values were constrained to be 1.5*U*_eq_ of the carrier atom for methyl H atoms and 1.2*U*_eq_ for the remaining H atoms.

### Crystal data

**Table:**

C_23_H_24_N_4_O_5_	*Z* = 2
*M**_r_* = 436.46	*F*(000) = 460
Triclinic, *P*1	*D*_x_ = 1.393 Mg m^−^^3^
Hall symbol: -P 1	Mo *K*α radiation, λ = 0.71073 Å
*a* = 9.3125 (1) Å	Cell parameters from 8871 reflections
*b* = 10.0941 (1) Å	θ = 2.5–32.6°
*c* = 12.9955 (2) Å	µ = 0.10 mm^−^^1^
α = 91.958 (1)°	*T* = 100 K
β = 107.752 (1)°	Block, orange
γ = 114.465 (1)°	0.34 × 0.20 × 0.13 mm
*V* = 1040.67 (2) Å^3^	

### Data collection

**Table:**

Bruker SMART APEXII CCD diffractometer	7466 independent reflections
Radiation source: fine-focus sealed tube	5375 reflections with *I* > 2σ(*I*)
graphite	*R*_int_ = 0.032
φ and ω scans	θ_max_ = 32.6°, θ_min_ = 1.7°
Absorption correction: multi-scan (*SADABS*; Bruker, 2009)	*h* = −14→14
*T*_min_ = 0.967, *T*_max_ = 0.987	*k* = −15→15
27734 measured reflections	*l* = −19→19

### Refinement

**Table:**

Refinement on *F*^2^	Primary atom site location: structure-invariant direct methods
Least-squares matrix: full	Secondary atom site location: difference Fourier map
*R*[*F*^2^ > 2σ(*F*^2^)] = 0.069	Hydrogen site location: inferred from neighbouring sites
*wR*(*F*^2^) = 0.197	H-atom parameters constrained
*S* = 1.04	*w* = 1/[σ^2^(*F*_o_^2^) + (0.0843*P*)^2^ + 0.8488*P*] where *P* = (*F*_o_^2^ + 2*F*_c_^2^)/3
7466 reflections	(Δ/σ)_max_ < 0.001
289 parameters	Δρ_max_ = 0.50 e Å^−^^3^
0 restraints	Δρ_min_ = −0.30 e Å^−^^3^

### Special details

**Table:**

Experimental. The crystal was placed in the cold stream of an Oxford Cryosystems Cobra open-flow nitrogen cryostat (Cosier & Glazer, 1986) operating at 100.0 (1) K.
Geometry. All esds (except the esd in the dihedral angle between two l.s. planes) are estimated using the full covariance matrix. The cell esds are taken into account individually in the estimation of esds in distances, angles and torsion angles; correlations between esds in cell parameters are only used when they are defined by crystal symmetry. An approximate (isotropic) treatment of cell esds is used for estimating esds involving l.s. planes.
Refinement. Refinement of F^2^ against ALL reflections. The weighted R-factor wR and goodness of fit S are based on F^2^, conventional R-factors R are based on F, with F set to zero for negative F^2^. The threshold expression of F^2^ > 2sigma(F^2^) is used only for calculating R-factors(gt) etc. and is not relevant to the choice of reflections for refinement. R-factors based on F^2^ are statistically about twice as large as those based on F, and R- factors based on ALL data will be even larger.

### Fractional atomic coordinates and isotropic or equivalent isotropic displacement parameters (Å^2^)

**Table:**

	*x*	*y*	*z*	*U*_iso_*/*U*_eq_	
O1	−0.29047 (16)	−0.18231 (15)	0.17443 (11)	0.0271 (3)	
O2	−0.45664 (18)	−0.31986 (16)	0.26240 (12)	0.0328 (3)	
O3	0.2053 (2)	0.51173 (19)	0.95685 (13)	0.0385 (4)	
O4	0.82578 (18)	0.95186 (18)	0.77738 (14)	0.0371 (4)	
O5	0.6533 (2)	1.04206 (17)	0.78208 (14)	0.0388 (4)	
N1	0.11744 (17)	0.29815 (16)	0.46007 (12)	0.0201 (3)	
N2	0.00661 (17)	0.29678 (16)	0.59278 (11)	0.0193 (3)	
N3	−0.0066 (2)	0.56743 (18)	0.86162 (13)	0.0245 (3)	
N4	0.6825 (2)	0.93979 (18)	0.75733 (13)	0.0268 (3)	
C1	−0.0276 (2)	0.16584 (19)	0.43862 (14)	0.0189 (3)	
C2	−0.1050 (2)	0.04589 (19)	0.35168 (14)	0.0201 (3)	
H2A	−0.0586	0.0455	0.2959	0.024*	
C3	−0.2518 (2)	−0.07262 (19)	0.34957 (14)	0.0208 (3)	
C4	−0.3197 (2)	−0.0732 (2)	0.43315 (15)	0.0233 (3)	
H4A	−0.4194	−0.1570	0.4299	0.028*	
C5	−0.2451 (2)	0.0444 (2)	0.51921 (14)	0.0224 (3)	
H5A	−0.2914	0.0443	0.5751	0.027*	
C6	−0.0981 (2)	0.16403 (19)	0.52027 (14)	0.0194 (3)	
C7	0.1333 (2)	0.37277 (18)	0.55203 (14)	0.0185 (3)	
C8	0.2713 (2)	0.52054 (19)	0.60439 (13)	0.0193 (3)	
C9	0.2460 (2)	0.6325 (2)	0.65125 (14)	0.0217 (3)	
H9A	0.1366	0.6135	0.6500	0.026*	
C10	0.3803 (2)	0.7712 (2)	0.69957 (14)	0.0221 (3)	
H10A	0.3638	0.8478	0.7310	0.026*	
C11	0.5388 (2)	0.79566 (19)	0.70104 (14)	0.0216 (3)	
C12	0.5674 (2)	0.6891 (2)	0.65223 (15)	0.0237 (3)	
H12A	0.6764	0.7100	0.6517	0.028*	
C13	0.4322 (2)	0.5512 (2)	0.60426 (14)	0.0221 (3)	
H13A	0.4491	0.4763	0.5708	0.027*	
C14	−0.3446 (2)	−0.2050 (2)	0.25940 (15)	0.0234 (3)	
C15	−0.3754 (2)	−0.3076 (2)	0.08290 (16)	0.0292 (4)	
H15A	−0.4990	−0.3501	0.0636	0.035*	
H15B	−0.3399	−0.3859	0.1032	0.035*	
C16	−0.3270 (3)	−0.2504 (3)	−0.01223 (19)	0.0441 (6)	
H16A	−0.3815	−0.3318	−0.0754	0.066*	
H16B	−0.2044	−0.2086	0.0080	0.066*	
H16C	−0.3632	−0.1734	−0.0316	0.066*	
C17	−0.0017 (2)	0.3308 (2)	0.70099 (13)	0.0214 (3)	
H17A	0.1063	0.4151	0.7467	0.026*	
H17B	−0.0149	0.2442	0.7376	0.026*	
C18	−0.1456 (2)	0.3693 (2)	0.69594 (14)	0.0236 (3)	
H18A	−0.2548	0.2819	0.6574	0.028*	
H18B	−0.1392	0.4503	0.6540	0.028*	
C19	−0.1347 (2)	0.4175 (2)	0.81236 (15)	0.0261 (4)	
H19A	−0.2453	0.4099	0.8095	0.031*	
H19B	−0.1099	0.3490	0.8594	0.031*	
C20	−0.0400 (3)	0.6949 (2)	0.84211 (18)	0.0354 (5)	
H20A	−0.1478	0.6790	0.8505	0.042*	
H20B	−0.0439	0.7153	0.7676	0.042*	
C21	0.1094 (4)	0.8213 (3)	0.9307 (2)	0.0443 (6)	
H21A	0.1467	0.9144	0.9017	0.053*	
H21B	0.0793	0.8379	0.9952	0.053*	
C22	0.2468 (3)	0.7692 (2)	0.96175 (18)	0.0353 (5)	
H22A	0.3248	0.8102	0.9210	0.042*	
H22B	0.3121	0.7996	1.0416	0.042*	
C23	0.1509 (2)	0.6021 (2)	0.92917 (15)	0.0268 (4)	

### Atomic displacement parameters (Å^2^)

**Table:**

	*U*^11^	*U*^22^	*U*^33^	*U*^12^	*U*^13^	*U*^23^
O1	0.0245 (6)	0.0248 (7)	0.0246 (6)	0.0055 (5)	0.0078 (5)	−0.0053 (5)
O2	0.0311 (7)	0.0247 (7)	0.0282 (7)	0.0022 (6)	0.0062 (6)	0.0020 (6)
O3	0.0361 (8)	0.0421 (9)	0.0348 (8)	0.0228 (7)	0.0031 (6)	−0.0081 (7)
O4	0.0244 (7)	0.0338 (8)	0.0417 (9)	0.0048 (6)	0.0088 (6)	0.0019 (7)
O5	0.0404 (8)	0.0261 (8)	0.0417 (9)	0.0100 (7)	0.0116 (7)	−0.0034 (6)
N1	0.0184 (6)	0.0202 (7)	0.0214 (7)	0.0080 (5)	0.0073 (5)	0.0017 (5)
N2	0.0188 (6)	0.0213 (7)	0.0165 (6)	0.0072 (5)	0.0070 (5)	0.0028 (5)
N3	0.0271 (7)	0.0251 (8)	0.0226 (7)	0.0122 (6)	0.0097 (6)	0.0024 (6)
N4	0.0276 (7)	0.0226 (8)	0.0231 (7)	0.0048 (6)	0.0089 (6)	0.0020 (6)
C1	0.0173 (7)	0.0202 (8)	0.0204 (7)	0.0086 (6)	0.0079 (6)	0.0034 (6)
C2	0.0194 (7)	0.0206 (8)	0.0200 (7)	0.0089 (6)	0.0068 (6)	0.0019 (6)
C3	0.0193 (7)	0.0205 (8)	0.0204 (7)	0.0079 (6)	0.0053 (6)	0.0028 (6)
C4	0.0199 (7)	0.0222 (8)	0.0240 (8)	0.0058 (6)	0.0078 (6)	0.0064 (6)
C5	0.0221 (7)	0.0228 (8)	0.0205 (7)	0.0069 (7)	0.0092 (6)	0.0048 (6)
C6	0.0188 (7)	0.0199 (8)	0.0178 (7)	0.0078 (6)	0.0055 (6)	0.0025 (6)
C7	0.0181 (7)	0.0177 (7)	0.0194 (7)	0.0078 (6)	0.0064 (6)	0.0028 (6)
C8	0.0195 (7)	0.0204 (8)	0.0174 (7)	0.0086 (6)	0.0063 (6)	0.0033 (6)
C9	0.0202 (7)	0.0230 (8)	0.0232 (8)	0.0105 (7)	0.0080 (6)	0.0037 (6)
C10	0.0264 (8)	0.0190 (8)	0.0207 (8)	0.0103 (7)	0.0080 (6)	0.0027 (6)
C11	0.0222 (7)	0.0194 (8)	0.0184 (7)	0.0051 (6)	0.0066 (6)	0.0028 (6)
C12	0.0200 (7)	0.0245 (9)	0.0241 (8)	0.0067 (7)	0.0093 (6)	0.0031 (7)
C13	0.0226 (8)	0.0240 (8)	0.0215 (8)	0.0104 (7)	0.0101 (6)	0.0031 (6)
C14	0.0208 (7)	0.0236 (8)	0.0225 (8)	0.0098 (7)	0.0036 (6)	0.0029 (6)
C15	0.0267 (9)	0.0276 (10)	0.0255 (9)	0.0091 (8)	0.0046 (7)	−0.0052 (7)
C16	0.0467 (13)	0.0415 (13)	0.0320 (11)	0.0082 (11)	0.0157 (10)	−0.0058 (10)
C17	0.0224 (7)	0.0263 (9)	0.0149 (7)	0.0103 (7)	0.0067 (6)	0.0033 (6)
C18	0.0196 (7)	0.0285 (9)	0.0202 (8)	0.0084 (7)	0.0072 (6)	−0.0003 (7)
C19	0.0234 (8)	0.0301 (9)	0.0238 (8)	0.0088 (7)	0.0119 (7)	0.0000 (7)
C20	0.0498 (12)	0.0293 (10)	0.0289 (10)	0.0226 (10)	0.0095 (9)	0.0032 (8)
C21	0.0650 (16)	0.0292 (11)	0.0329 (11)	0.0212 (11)	0.0094 (11)	0.0030 (9)
C22	0.0365 (10)	0.0305 (10)	0.0285 (10)	0.0038 (9)	0.0143 (8)	−0.0019 (8)
C23	0.0284 (9)	0.0302 (10)	0.0216 (8)	0.0116 (8)	0.0114 (7)	−0.0017 (7)

### Geometric parameters (Å, °)

**Table:**

O1—C14	1.337 (2)	C10—C11	1.386 (2)
O1—C15	1.461 (2)	C10—H10A	0.9500
O2—C14	1.208 (2)	C11—C12	1.387 (3)
O3—C23	1.229 (3)	C12—C13	1.386 (3)
O4—N4	1.231 (2)	C12—H12A	0.9500
O5—N4	1.227 (2)	C13—H13A	0.9500
N1—C7	1.325 (2)	C15—C16	1.496 (3)
N1—C1	1.392 (2)	C15—H15A	0.9900
N2—C6	1.380 (2)	C15—H15B	0.9900
N2—C7	1.386 (2)	C16—H16A	0.9800
N2—C17	1.468 (2)	C16—H16B	0.9800
N3—C23	1.347 (2)	C16—H16C	0.9800
N3—C19	1.448 (2)	C17—C18	1.527 (2)
N3—C20	1.456 (3)	C17—H17A	0.9900
N4—C11	1.470 (2)	C17—H17B	0.9900
C1—C2	1.396 (2)	C18—C19	1.534 (2)
C1—C6	1.405 (2)	C18—H18A	0.9900
C2—C3	1.386 (2)	C18—H18B	0.9900
C2—H2A	0.9500	C19—H19A	0.9900
C3—C4	1.413 (2)	C19—H19B	0.9900
C3—C14	1.492 (2)	C20—C21	1.531 (3)
C4—C5	1.377 (3)	C20—H20A	0.9900
C4—H4A	0.9500	C20—H20B	0.9900
C5—C6	1.396 (2)	C21—C22	1.527 (4)
C5—H5A	0.9500	C21—H21A	0.9900
C7—C8	1.466 (2)	C21—H21B	0.9900
C8—C13	1.400 (2)	C22—C23	1.515 (3)
C8—C9	1.402 (2)	C22—H22A	0.9900
C9—C10	1.389 (3)	C22—H22B	0.9900
C9—H9A	0.9500		
			
C14—O1—C15	115.53 (15)	O1—C14—C3	112.44 (15)
C7—N1—C1	104.74 (14)	O1—C15—C16	107.13 (17)
C6—N2—C7	106.18 (13)	O1—C15—H15A	110.3
C6—N2—C17	124.31 (14)	C16—C15—H15A	110.3
C7—N2—C17	128.43 (14)	O1—C15—H15B	110.3
C23—N3—C19	123.98 (17)	C16—C15—H15B	110.3
C23—N3—C20	114.20 (17)	H15A—C15—H15B	108.5
C19—N3—C20	121.81 (16)	C15—C16—H16A	109.5
O5—N4—O4	123.33 (17)	C15—C16—H16B	109.5
O5—N4—C11	118.19 (16)	H16A—C16—H16B	109.5
O4—N4—C11	118.48 (16)	C15—C16—H16C	109.5
N1—C1—C2	129.65 (15)	H16A—C16—H16C	109.5
N1—C1—C6	110.09 (15)	H16B—C16—H16C	109.5
C2—C1—C6	120.25 (16)	N2—C17—C18	113.73 (14)
C3—C2—C1	117.63 (16)	N2—C17—H17A	108.8
C3—C2—H2A	121.2	C18—C17—H17A	108.8
C1—C2—H2A	121.2	N2—C17—H17B	108.8
C2—C3—C4	121.30 (16)	C18—C17—H17B	108.8
C2—C3—C14	121.58 (16)	H17A—C17—H17B	107.7
C4—C3—C14	117.12 (16)	C17—C18—C19	110.22 (14)
C5—C4—C3	121.73 (16)	C17—C18—H18A	109.6
C5—C4—H4A	119.1	C19—C18—H18A	109.6
C3—C4—H4A	119.1	C17—C18—H18B	109.6
C4—C5—C6	116.66 (16)	C19—C18—H18B	109.6
C4—C5—H5A	121.7	H18A—C18—H18B	108.1
C6—C5—H5A	121.7	N3—C19—C18	112.88 (15)
N2—C6—C5	131.67 (16)	N3—C19—H19A	109.0
N2—C6—C1	105.92 (14)	C18—C19—H19A	109.0
C5—C6—C1	122.42 (16)	N3—C19—H19B	109.0
N1—C7—N2	113.07 (15)	C18—C19—H19B	109.0
N1—C7—C8	122.98 (15)	H19A—C19—H19B	107.8
N2—C7—C8	123.96 (15)	N3—C20—C21	103.43 (18)
C13—C8—C9	119.31 (16)	N3—C20—H20A	111.1
C13—C8—C7	118.47 (15)	C21—C20—H20A	111.1
C9—C8—C7	122.19 (15)	N3—C20—H20B	111.1
C10—C9—C8	120.27 (15)	C21—C20—H20B	111.1
C10—C9—H9A	119.9	H20A—C20—H20B	109.0
C8—C9—H9A	119.9	C22—C21—C20	104.44 (18)
C11—C10—C9	118.67 (16)	C22—C21—H21A	110.9
C11—C10—H10A	120.7	C20—C21—H21A	110.9
C9—C10—H10A	120.7	C22—C21—H21B	110.9
C10—C11—C12	122.60 (16)	C20—C21—H21B	110.9
C10—C11—N4	118.71 (16)	H21A—C21—H21B	108.9
C12—C11—N4	118.68 (16)	C23—C22—C21	104.12 (18)
C13—C12—C11	118.05 (16)	C23—C22—H22A	110.9
C13—C12—H12A	121.0	C21—C22—H22A	110.9
C11—C12—H12A	121.0	C23—C22—H22B	110.9
C12—C13—C8	121.02 (16)	C21—C22—H22B	110.9
C12—C13—H13A	119.5	H22A—C22—H22B	109.0
C8—C13—H13A	119.5	O3—C23—N3	124.88 (19)
O2—C14—O1	123.64 (17)	O3—C23—C22	127.05 (19)
O2—C14—C3	123.92 (17)	N3—C23—C22	108.06 (18)
			
C7—N1—C1—C2	179.48 (17)	C9—C10—C11—N4	−176.78 (16)
C7—N1—C1—C6	0.23 (19)	O5—N4—C11—C10	−13.9 (3)
N1—C1—C2—C3	−179.08 (17)	O4—N4—C11—C10	166.00 (17)
C6—C1—C2—C3	0.1 (2)	O5—N4—C11—C12	166.58 (18)
C1—C2—C3—C4	−0.9 (3)	O4—N4—C11—C12	−13.5 (3)
C1—C2—C3—C14	179.30 (15)	C10—C11—C12—C13	−2.7 (3)
C2—C3—C4—C5	1.2 (3)	N4—C11—C12—C13	176.81 (16)
C14—C3—C4—C5	−179.04 (16)	C11—C12—C13—C8	0.4 (3)
C3—C4—C5—C6	−0.5 (3)	C9—C8—C13—C12	1.7 (3)
C7—N2—C6—C5	−179.83 (18)	C7—C8—C13—C12	179.80 (16)
C17—N2—C6—C5	11.2 (3)	C15—O1—C14—O2	−0.5 (3)
C7—N2—C6—C1	0.53 (18)	C15—O1—C14—C3	179.81 (15)
C17—N2—C6—C1	−168.39 (15)	C2—C3—C14—O2	167.84 (18)
C4—C5—C6—N2	−179.88 (17)	C4—C3—C14—O2	−12.0 (3)
C4—C5—C6—C1	−0.3 (3)	C2—C3—C14—O1	−12.4 (2)
N1—C1—C6—N2	−0.49 (19)	C4—C3—C14—O1	167.77 (15)
C2—C1—C6—N2	−179.81 (15)	C14—O1—C15—C16	167.07 (17)
N1—C1—C6—C5	179.83 (16)	C6—N2—C17—C18	−77.5 (2)
C2—C1—C6—C5	0.5 (3)	C7—N2—C17—C18	116.10 (19)
C1—N1—C7—N2	0.12 (19)	N2—C17—C18—C19	−174.58 (15)
C1—N1—C7—C8	−179.84 (15)	C23—N3—C19—C18	−95.5 (2)
C6—N2—C7—N1	−0.42 (19)	C20—N3—C19—C18	85.1 (2)
C17—N2—C7—N1	167.89 (16)	C17—C18—C19—N3	77.3 (2)
C6—N2—C7—C8	179.53 (15)	C23—N3—C20—C21	−13.5 (2)
C17—N2—C7—C8	−12.2 (3)	C19—N3—C20—C21	165.93 (18)
N1—C7—C8—C13	−38.3 (2)	N3—C20—C21—C22	22.3 (2)
N2—C7—C8—C13	141.76 (17)	C20—C21—C22—C23	−23.2 (2)
N1—C7—C8—C9	139.76 (18)	C19—N3—C23—O3	0.2 (3)
N2—C7—C8—C9	−40.2 (2)	C20—N3—C23—O3	179.69 (19)
C13—C8—C9—C10	−1.7 (3)	C19—N3—C23—C22	179.15 (16)
C7—C8—C9—C10	−179.69 (16)	C20—N3—C23—C22	−1.4 (2)
C8—C9—C10—C11	−0.5 (3)	C21—C22—C23—O3	−165.3 (2)
C9—C10—C11—C12	2.7 (3)	C21—C22—C23—N3	15.8 (2)

### Hydrogen-bond geometry (Å, °)

**Table:**

*D*—H···*A*	*D*—H	H···*A*	*D*···*A*	*D*—H···*A*
C15—H15A···O3^i^	0.99	2.41	3.355 (3)	159
C15—H15B···O3^ii^	0.99	2.38	3.186 (3)	139
C19—H19A···O2^iii^	0.99	2.38	3.312 (3)	156

Symmetry codes: (i) *x*−1, *y*−1, *z*−1; (ii) −*x*, −*y*, −*z*+1; (iii) −*x*−1, −*y*, −*z*+1.
